# GAPDH: is it a reliable housekeeper gene to use in sepsis research?

**DOI:** 10.1186/cc11794

**Published:** 2012-11-14

**Authors:** J Sarveswaran, NM Orsi, M Cummings, S Homer-Vanniasinkam, D Burke

**Affiliations:** 1 Leeds Teaching Hospitals, Leeds, UK

## Background

Quantitative gene expression data are often normalised to the expression levels of control genes (that is, housekeeper genes). An assumption in the use of housekeeper genes is that the expression of the genes remains constant in the cells/tissues under investigation. Glyceraldehyde-3-phosphate dehydrogenase (GAPDH) is one of the most commonly used housekeeping genes used in comparisons of gene expression data. Recent studies, however, have shown that GAPDH expression is highly variable in different experimental settings. This study aimed to characterise GAPDH mRNA expression in severe sepsis, using whole blood from patients with severe sepsis and healthy controls.

## Methods

Patients admitted to the ICU within 24 hours of onset of severe sepsis were recruited. Healthy controls were also recruited. Exclusion criteria included patients on immunosuppressants or chemotherapy and those with a moribund status. Ethical approval (REC W06/Q1107/42) was obtained. Whole blood (1 ml) was taken from the patients and Quantigene RNA extraction techniques were used. The Quantigene plex assay was used in duplicate to determine GAPDH mRNA expression. The assay uses branched DNA signal amplification to enable the detection and quantification of multiple RNA targets simultaneously. It has been compared with other techniques favourably [1]. GAPDH mRNA expression was standardised to housekeeper genes succinate dehydrogenase (SDHA) and hypoxanthine-guanine phosphoribosyltransferase (HPRT).

## Results

Sixty-five patients with severe sepsis were included, of whom 27 died within 28 days. Seventeen control samples were included in the study. The groups were age and sex matched. In terms of GAPDH mRNA expression there was no significant difference between survivors and nonsurvivors. However, with regards to overall GAPDH mRNA expression in severe sepsis there was a marked increase in patients with severe sepsis. GAPDH mRNA expression showed a 16-fold mean increase in expression compared with control samples.

## Conclusion

GAPDH mRNA expression in patients with severe sepsis showed a marked increase compared with controls. These data question the suitability of GAPDH as a housekeeper gene in gene expression profiling studies in sepsis. The data also highlight GAPDH as another potential biomarker of sepsis that requires further evaluation.
